# Stat3 and CCAAT/enhancer binding protein beta (C/EBP-beta) regulate Jab1/CSN5 expression in mammary carcinoma cells

**DOI:** 10.1186/bcr2902

**Published:** 2011-06-20

**Authors:** Terry J Shackleford, Qingxiu Zhang, Ling Tian, Thuy T Vu, Anita L Korapati, Angela M Baumgartner, Xiao-Feng Le, Warren S Liao, Francois X Claret

**Affiliations:** 1 Department of Systems Biology, Unit 950, University of Texas - MD Anderson Cancer Center, 1515 Holcombe Boulevard, Houston, TX 77030, USA; 2 Department of Experimental Therapeutics, University of Texas - MD Anderson Cancer Center, 1515 Holcombe Boulevard, Houston, TX 77030, USA

## Abstract

**Introduction:**

The c-Jun coactivator, Jun activation-domain binding protein 1 (Jab1) also known as the fifth component of the COP9 signalosome complex (CSN5), is a novel candidate oncogene whose aberrant expression contributes to the progression of breast carcinoma and other human cancers. The mechanism of *Jab1 *gene expression and its deregulation in cancer cells remains to be identified. We therefore investigated the transcriptional regulatory mechanisms of *Jab1 *expression in human breast carcinoma cells.

**Methods:**

To identify potential regulators of *Jab1 *transcription, we cloned the 5' upstream region of the human *Jab1 *gene and mapped its transcriptional start site. We identified binding sequences for the CCAAT/enhancer binding protein (C/EBP) and GATA, as well as a signal transducer and activator of transcription-3 (Stat3) consensus sequence overlapping the C/EBP site, using 5'- deletion analysis and a gene reporter assay. Mutational analysis of these binding sites was performed to confirm their roles in promoting *Jab1 *transcription in breast cancer cells. We further confirmed these binding sites using electrophoretic mobility shift assays (EMSAs) and chromatin immunoprecipitation (ChIP) assays. We also analyzed whether the siRNA-mediated inactivation of Stat3 and Src could reduce *Jab1*-promoter activity and whether interleukine-6 (IL-6) could mediate increased Jab1 expression through Stat3 signaling.

**Results:**

We identified binding sequences for C/EBP, GATA, as well as a Stat3 consensus sequence overlapping the C/EBP site in the promoter region of *Jab1*. C/EBP-beta2 is a potential transcriptional activator of *Jab1 *and mutation of the C/EBP/Stat3 binding site significantly reduced *Jab1*-promoter activity. In addition, inhibiting Stat3 significantly reduced *Jab1*-promoter activation. EMSA and ChIP assays confirmed that C/EBP, GATA1 and Stat3 bind to Jab1 promoter in breast carcinoma cells. We also found that Src, an activator of Stat3, is involved in *Jab1*-promoter activation. siRNA knockdown of Src reduced the *Jab1*-promoter activity, similar to the results seen when Stat3 was inhibited in breast carcinoma cells. Interestingly, reactivation of Stat3 in normal mammary epithelial cells (MCF-10A, MCF-10F) is sufficient to reactivate *Jab1 *expression. Treatment with the cytokine IL-6 resulted in increased Jab1 expression that was blocked by inhibition of Stat3.

**Conclusions:**

These findings reveal a novel mechanism of *Jab1 *gene regulation and provide functional and mechanistic links between the Src/Stat3 and IL-6/Stat3 signaling axes that are involved in the activation of *Jab1 *transcription and regulation of this novel oncogenic protein.

## Introduction

c-Jun activation domain-binding protein-1 (Jab1) is a multifunctional protein that regulates cell proliferation and oncogenesis. Since its identification as a c-Jun coactivator [1], Jab1 has been found to be an integral component of the COP9 signalosome (CSN) complex, a multifunctional protein complex involved in modulating signal transduction, gene transcription, and protein stability [2-4]. Jab1 is the fifth subunit of the CSN and is also referred to as CSN5. One of the most recognized functions of the CSN is the deneddylation of the cullin-RING-ubiquitin ligase (CRL) and this function is reliant on the Jab1 MPN domain metalloenzyme (JAMM) motif that serves as the catalytic center [5,6]. Jab1 exists not only as a member of the CSN holocomplex and smaller CSN complexes, but also as a monomer with a number of different unique protein interactions and functions outside of the CSN.

Jab1 functionally inactivates several key negative regulatory proteins by affecting their subcellular localization, degradation, phosphorylation, and deneddylation, thereby acting as a positive regulator of cellular proliferation. Through these interactions, Jab1 plays a crucial role in the inactivation of several key tumor suppressors, including cyclin-dependent kinase inhibitor p27^Kip1^, p53, and Smad4/7 [7-10]. It can also interact with several important intracellular signaling molecules including hypoxia inducible factor-1 alpha (HIF-1α), macrophage migration inhibitory factor (MIF), E2F1, and cullin 1 (CUL-1) [11,12].

Abnormal overexpression of Jab1 has been detected in several types of cancer in humans and in some cases correlates with poor prognosis and low-level expression of p27 [13-18]. However, the molecular mechanism for up-regulation of Jab1 in cancer cells is still unclear. Our studies have shown that Jab1 and p27 protein levels are inversely correlated in invasive breast carcinoma specimens and that Jab1 is highly expressed in breast tumor samples relative to paired normal-tissue samples [14]. Jab1, along with the oncogene Myc, reside on the frequently amplified region on chromosome 8 and were identified to induce a wound signature in human breast cancer cells [19]. Further investigations identified the isopeptidase activity of Jab1 to be critical for its ability to promote transformation and progression in breast epithelial cells and inhibition of this activity is sufficient to block breast cancer progression driven by MYC and RAS [20]. These findings suggest that Jab1 is an important regulator in cancer development and preclinical studies suggest that inhibition of Jab1 delays tumor growth [14].

Given the frequency of Jab1 overexpression in human cancers and its potential role in the development of cancer, identifying the mechanism by which Jab1 overexpression occurs would be of great interest. The extent to which Jab1 amplification on chromosome 8q is responsible for its frequent overexpression in cancer has not yet been investigated. However, additional mechanisms of regulation through transcriptional control are likely to also be of importance and may link its regulation to upstream signaling pathways. We hypothesize that overexpression of Jab1 in breast cancer can be attributed to an increase in transcriptional activity over that seen in normal tissue. We therefore studied the transcriptional regulation of Jab1 in breast cancer cells. In this present study, we describe the cloning and characterization of the human *Jab1 *promoter. We also identify a region whereby CCAAT/enhancer binding protein-beta (C/EBP-β), signal transducer and activator of transcription-3 (Stat3), and GATA1 induce *Jab1 *transcription and identify a potential upstream oncogenic signaling molecule that may be key to the regulation of Jab1 expression in cancer. The region we describe here has also recently been identified by another group to contain a T-cell transcription factor (TCF)-4 and Sp1 binding site that was found to be important for transcription and activated by human epidermal growth factor receptor (HER)-2 activation of the AKT/β-catenin pathway [21], which points to the importance of this region in driving the transcription of *Jab1 *and possibly linking its expression to potent oncogenic signaling pathways.

## Materials and methods

### Cell lines, reagents and antibodies

The human breast cancer cell lines, MCF7, MDA-MB-468, MDA-MB-231, BT-474, ZR-75-1, BT-549, MDA-MB-453, T47D and non-tumorigenic human breast epithelial MCF-10A, MCF-10F, HMEC, and 184A, were purchased from the American Type Culture Collection (Manassas, VA, USA). None were derived directly from tumor tissue for the purposes of this study. MCF7 cells were grown in DMEM. Breast cancer cells were grown in RPMI medium supplemented with 10% FBS. MCF-10A and MCF-10F cells were grown in 50% DMEM, 50% F-12 medium supplemented with 5% horse serum, 100 units/ml penicillin, 100 μg/ml streptomycin, 10 μg/ml insulin, 100 ng/ml cholera toxin, 0.5 μg/ml hydrocortisone, 20 ng/ml recombinant human epidermal growth factor, and 1 mM CaCl_2_. The following antibodies were used in the study: C/EBP-α (N-19), C/EBP-β (C-19), GATA-1 (H-200), and Stat3 (C-20) (Santa Cruz Biotechnology, Santa Cruz, CA, USA). The following antibodies were obtained from Cell Signaling (Danvers, MA, USA): Src, Phospho-Stat3(Y705), β-Tubulin, and β-actin. Anti-Flag was obtained from Sigma-Aldrich (St. Louis, MO, USA). IL-6 was obtained from Invitrogen (Carlsbad, CA, USA) and used at 40 ng/mL. The Stattic inhibitor (Sigma, St. Louis, MO, USA) was used at 20 nM.

### Primer extension

An antisense primer, P1 was designed 5' of the ATG translation site of the *Jab1 *gene and end-labeled with T4 polynucleotide kinase and ^32^P-γ-ATP, followed by purification using Nu-Clean D25 columns (Shelton Scientific-IBI, Peosta, IA, USA). Total RNA was isolated from MDA-MD-231 cells using TRIzol reagent (Invitrogen, Carlsbad, CA, USA), and 20 μg of RNA was hybridized with 1 × 10^6 ^c.p.m. of ^32^P-labeled P1 oligonucleotide. The protocol for the Primer Extension System-AMV Reverse Transcriptase (Promega, Madison, WI, USA) was followed. The size of the extended product was determined by electrophoresis on a 6% polyacrylamide gel containing 6 M urea. To determine the size of the primer extended fragment, the labeled Promega marker and a sequencing reaction with *Jab1 *cDNA as template and primer P1 using the SequiTherm Excel II DNA Sequencing Kit (Epicentre, Madison, WI, USA) were run simultaneously on the gel.

### Computer analysis

Transcription factor binding sites were predicted using Genomatix software (Munich, Germany); the MatInspector program, which uses TRANSFAC matrices; and the WebGene HCtata program's Hamming clustering method for TATA signal prediction in eukaryotic genes.

### Cloning and analysis of the human Jab1 promoter

To clone the 5'-flanking region of the human *Jab1 *gene, a bacterial artificial chromosome clone containing the region corresponding to *Jab1 *was used as a template for PCR. The amplified DNA fragments were subcloned into the luciferase reporter vector pGL3 (Promega, Madison, W, USA). Progressive deletion mutants of the pGL3-Jab1 promoter were created by PCR. The integrity of constructs was confirmed by DNA sequencing. The following primers were used: +83R, -2006F, -2958F, -946F, -658F, -472F, -344F, -127F, and -59F (Table 1).

**Table 1:** Sequences of oligonucleotides used in RT-PCR, primer extension, cloning site- directed mutagenesis, EMSA and ChIP

Oligonucleotide	Sequence (5' à 3')	Purpose
Jab1 F296-314	ATCTCAGCATTGGCTCTGC	RT-PCR
Jab1 R1094-1076	TAACCTGAGACATCAATC	RT-PCR
Jab1 R883-864	AGAACTCAACGTATTCACCC	RT-PCR
GAPDH F	CCACCCATGGCAAATTCCATGGCA	RT-PCR
GAPDH R	TCTAGACGGCAGGTCAGGTCCACC	RT-PCR
P1	CGCTCCCGGACGCCGCC	Primer extension
+83R	CTGAAAGCTTCGCTCCCGGACGCCGCC	Cloning
-2006F	CTGACTCGAGGTGGTAGTGAGAGG	Cloning
-2958F	CTGACTCGAGCGCATGGGAACCAA	Cloning
-946F	TGAACTCGAGCCTGCTCCCTGTGTC	Cloning
-658F	CTGACTCGAGCCCACTGCCTCCTCG	Cloning
-472F	CTGAGCTAGCCAACAGACAGCCTT	Cloning
-344F	CTGACTCGAGGAGGCCGAGCCTGC	Cloning
-127F	TGAGCTAGCGTCCCGGAAAGGTCCCC	Cloning
-59F	TGACTCGAGCTGCCCCAAGAGTC	Cloning
-31F	CTGAGCTAGCGTTCCCGTGGTGCGG	Cloning
CEBP-Mut	GCCTTACCTTTTAGTCTTTCctgAAACTTATCTC	Site-directed mutagenesis
GATA1-Mut	CAACAAACTTgagTCATTTAAGGTACCTATACCC	Site-directed mutagenesis
GATA1-Del	GTCTTTCAACAAACTT....CATTTAAGGTACC	Site-directed mutagenesis
-462/-445 CEBP WT	CCTTACCTTTTAGTCTTTCAACAAACT	EMSA
-462/-445 CEBP Mut	CCTTACCTTTTAGTCTTTCctgcAAACT	EMSA
-444/-417 GATA1 WT	AATCATTTATCTCATTTAAGGTACC	EMSA
-444/-417 GATA1 M1	GGAGTCATTTAAGGTACCTATACCC	EMSA
-472F	CTGACTCGAGCAACAGACAGC	ChIP
-343R	CTGAAAGCTTCGCAGGCT	ChIP

Lower-case letters represent mutated oligonucleotides.

ChIP, chromatin immunoprecipitation; Del, deletion; EMSA, electrophoretic mobility shift assay; F, forward; Mut, mutant; M1, mutant-1; P, primer; R, reverse; RT-PCR, reverse-transcription polymerase chain reaction; WT, wild-type.

### PCR and RT-PCR

The PCR reaction contained 100 ng of DNA template, 1.5 mM MgCl_2_, 0.2 mM dNTP (Roche Applied Science, Indianapolis, IN, USA), 1 uM of primers, and Taq High Fidelity DNA polymerase (Invitrogen, Carlsbad, CA, USA).

The reverse transcriptase (RT) assay was performed from 2 μg of total RNA using Superscript II RT (Invitrogen, Carlsbad, CA, USA) according to the manufacturer's procedure. A reaction without RT was performed in parallel to ensure the absence of genomic DNA contamination. PCR amplification was carried out as described previously [22]. Conditions for the PCR reaction consisted of an initial denaturation step at 94°C for 5 minutes, followed by 30 cycles of 30 seconds at 94°C, 30 seconds at 60°C, and 30 seconds at 68°C. After a final extension at 72°C for 10 minutes, PCR products were resolved on 1.2% agarose gels and visualized by ethidium bromide transillumination under UV light. Primers used were: *Jab1 *F296-314, *Jab1 *R1094-1076, *Jab1 *R883-864, *GAPDH *F, and *GAPDH *R. For these and all following primer sequences please refer to Table 1.

### Transient transfection with reporter constructs and luciferase assay

MCF7, MDA-MB-231, and MDA-MB-468 cells were plated into 24-well tissue culture dishes at 4 × 10^4 ^cells/well 24 hours before transfection. Transfections were performed in triplicate according to the manufacturer's protocol using Lipofectamine PLUS reagent (Invitrogen, Carlsbad, CA, USA). Briefly, 0.4 μg reporter plasmid Jab1-Luc (Firefly luciferase) together with 10 ng of pRL (*Renilla *luciferase) were cotransfected. Luciferase assays were performed 36 hours after transfection using a Dual-Luciferase Reporter Assay System (Promega, Madison, W, USA). Firefly and *Renilla *luciferase activities were read on a Monolight 3010 luminometer (BD Bioscience, Rockville, MD, USA). Firefly luciferase activity was normalized to *Renilla *luciferase readings in each well. Each experiment was conducted at least twice in triplicate.

### Mutagenesis of the Jab1 promoter

Site-directed mutagenesis of *CEBP *and *GATA1 *was performed according to the QuickChange II method (Stratagene, La Jolla, CA, USA). The following mutagenic primers were used: *CEBP-Mut*, *GATA1-Mut*, *and GATA1-Del *(see Table 1 for sequences). All mutants were verified by sequencing.

### Nuclear extract preparation and electrophoretic mobility shift assay

Nuclear extracts were prepared as previously described [1]. Briefly, MCF7 and MDA-MB-468 cells were lysed in 10 mM HEPES-KOH (pH 7.9), 10 mM KCl, 0.1 mM EDTA, 0.1 mM EGTA, 1 mM DTT, and protease inhibitor cocktail (Roche Applied Science, Indianapolis, IN, USA). After incubation for 10 minute on ice, nuclei were recovered by centrifugation at 3,000 × *g *at 4°C for one minute and resuspended in 20 mM HEPES-KOH (pH 7.9), 0.4 M NaCl, 1 mM EDTA, 1 mM EGTA, 1 mM DTT, and protease inhibitor cocktail. Protein concentrations were determined using the DC Protein Assay (Bio-Rad Laboratories, Hercules, CA, USA). The following double-stranded DNA oligonucleotides were used in the electrophoretic mobility shift assays (EMSAs): -462/-436 CEBP-WT, -462/-436 CEBP-M1, -435/-417 GATA1-WT, and -435/-417 GATA1-M1 (see Table 1 for sequences).

Oligonucleotides were end-labeled with [γ-^32^P] ATP (MP Biomedicals, Solon, OH, USA) by T4 polynucleotide kinase (New England Biolabs, Ipswich, MA, USA) and purified with Quick Spin G-50 Columns (Roche Applied Science, Indianapolis, IN, USA). Pre-binding of 5 μg of nuclear extract to 0.05 mg/mL poly(dI:dC) (Roche, Applied Science, Indianapolis, IN, USA) was performed in a buffer containing 20 mM HEPES (pH 7.4), 0.1 mM EDTA (pH 8.0), 75 mM KCl, 2.5 mM MgCl_2_, 1 mM DTT, and 5% glycerol for 20 minutes at room temperature (22 to 24°C) before addition of 60,000 c.p.m. of labeled probe. For competition assays, 25- and 100-fold excess cold competitor oligonucleotide duplex was added to the reaction buffer 10 minutes before addition of the labeled probes. For supershift assays, antibodies were added for 20 minutes at 4°C prior to addition of the labeled probe. Reactions were resolved by electrophoresis on a 4.5% nondenaturing polyacrylamide gel run in 0.5 × TBE, vacuum-dried with heat, and exposed to film at -80°C.

### Chromatin immunoprecipitation (ChIP) assay

The manufacturer's protocol for the chromatin immunoprecipitation (ChIP) Assay Kit (Upstate Biotechnology, Temecula, CA, USA) was followed. Briefly, MCF7 cells or MDA-MB-231 cells transfected with control pcDNA vector or C/EBP-β2 and were incubated with 1% formaldehyde for 20 minutes at 37°C. Cells were collected, lysed, sonicated, and incubated with 4 μg of antibodies to C/EBP-α, C/EBP-β, GATA-1, Stat3, or β-actin overnight. PCR was used to amplify DNA bound to the immunoprecipitated histones after reversing the histone-DNA cross-links. The following primers were used for PCR: -472F and -344R (see Table 1 for sequences).

### Transfection of small interfering RNA oligonucleotides

Small interfering RNA (siRNA) for *Stat3*, *Src*, and Control (LUC) were obtained from Dharmacon (Lafayette, CO, USA). Oligonucleotides were transfected using Oligofectamine (Invitrogen, Carlsbad, CA, USA) following the manufacturer's protocol. For luciferase assay experiments, MDA-MB-468 cells were plated at 4 × 10^4 ^cells per well in a 24-well tissue culture dish. siRNA (20 nmol and 50 nmol) were transfected in complete medium without antibiotics. The -472 Jab1-Luc construct and pRL were cotransfected 24 hours later using the manufacturer's protocol for Lipofectamine PLUS transfection reagent. Luciferase assays were performed after 48 hours.

## Results

### Mapping the transcription initiation start site of the human Jab1 gene

**Figure 1:**
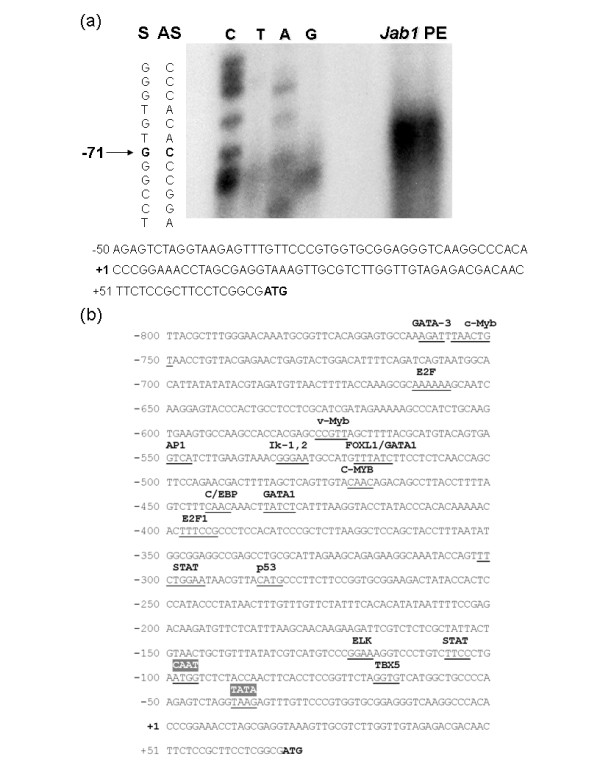
**Identification of the transcriptional start site and promoter region of the human *Jab1 *gene**. (**a**) Primer extension (PE) analysis was performed using an oligonucleotide corresponding to the antisense sequence at the translational start site (+1). A sequencing reaction using the same primer labeled with [γ -^32^P] ATP was included as a size marker and is shown on the left. An extension product of 71 bp was observed. (**b**) Sequence of the promoter region of *Jab1*, with potential regulatory elements (underlined) identified according to the TRANSFAC databases. bp, base pairs; Jab1, c-Jun activation domain-binding protein-1.

To determine the transcription start site of the *Jab1 *gene, primer extension analysis was performed. An antisense oligonucleotide (P1 primer) was synthesized corresponding to the sequence located at the ATG translational start site according to the published sequence [GenBank accession no. NT008183]. The P1 primer was radio-labeled and extended using avian myeloblastosis virus reverse transcriptase and analyzed on a polyacrylamide-urea gel along with a DNA cycle sequencing reaction using the same primer. The primer extension experiment revealed an extension product of 71 nucleotides using the P1 primer (Figure 1a). The nucleotide 71 bp upstream of the ATG translation start site was designated the +1 transcription initiation site in the numbering of the nucleotide sequence throughout this study.

### Identification of the transcription elements located in the Jab1 promoter

Based on the determined location of the transcription start site, we analyzed the *Jab1 *5' flanking region for a functional promoter. Using the MatInspector program, which uses TRANSFAC transcription factor binding site matrices, we identified a number of putative transcription factor binding sites upstream of the *Jab1 *transcription start site (Figure 1b). A typical TATA box [23] was found 40 bp upstream of the transcription start site, along with a CAAT box at -99 bp. A number of putative transcription factor-binding elements were present in the promoter sequence, including STAT, ELK, p53, E2F, C/EBP, GATA, c-Myb, and AP-1.

### Identification of Cis-Acting elements within the Jab1 gene promoter

**Figure 2:**
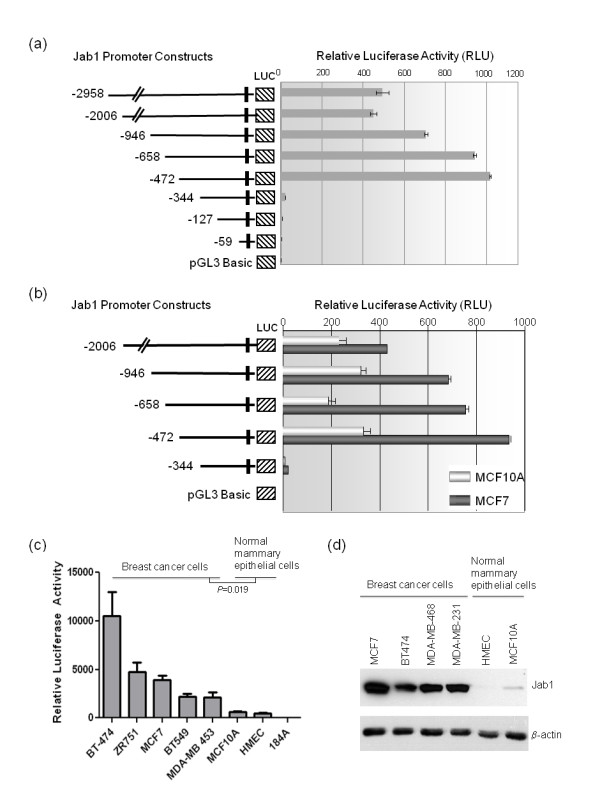
**5' deletion of the *Jab1 *promoter analysis reveals increased activity in breast cancer cells compared with normal cells**. (**a**) Identification of the minimal promoter region of *Jab1 *using progressive deletion of the 5' region in luciferase reporter constructs. The plasmids were cotransfected with *Renilla *luciferase plasmid pRL-null (transfection control) into MCF7 cells, and luciferase activity was assayed. Values represent mean ± standard error of triplicate experiments. Luciferase assays shown in subsequent figures were performed similarly. (**b**) *Jab1 *promoter 5' deletion constructs were transfected into MCF7 and MCF-10A cells and subjected to luciferase reporter assays. Promoter activity was higher in MCF7 epithelial breast cancer cells than in non-tumorigenic epithelial MCF-10A cells. Deletion of the region -472 to -344 resulted in a loss of promoter activity in both cell lines. (**c**) A panel of breast cancer cells and normal mammary cells were transfected with the -472 Jab1-Luc construct. Significance is noted as * *P *< 0.05 and ** *P *< 0.01 and was determined by t-test. (**d**) Jab1 protein levels for the cell lines in (c) were detected by western blotting, β-actin is shown as a control. Jab1, c-Jun activation domain-binding protein-1.

To analyze the mechanisms responsible for transcriptional regulation of *Jab1*, luciferase constructs were generated to evaluate *Jab1 *promoter activity. A fragment of the human *Jab1 *promoter region from -2958 to +68 was amplified, sequenced, and subcloned into the luciferase reporter vector pGL3. To identify regulatory sequences that are important for transcriptional control of the *Jab1 *gene, we created a series of 5'-deletion constructs of the full length *Jab1 *promoter and analyzed for their ability to drive the expression of luciferase in MCF7 breast cancer cells (Figure 2a). Serial 5'-deletion mutations of the full-length promoter revealed a pattern of functional activity in transfected cells. The results suggest that the core promoter elements that drive maximal promoter activity are within the sequence spanning -472 and -345 upstream of the transcription initiation site, as the -344 Jab1-Luc construct displayed a marked loss of promoter activity (Figure 2a). Similar patterns of activity were seen in other breast cancer cell lines including BT-549, MDA-MB-231, SKBR3, and BT-474 (data not shown). Additionally, we evaluated the promoter strength in mammary tumor cells versus non-tumor cells. *Jab1 *promoter activity was three times higher in MCF7 breast cancer cells when observing the -472 construct than in MCF-10A normal mammary epithelial cells (Figure 2b). Further analysis of a panel of breast cancer cell lines including BT-474, ZR-75-1, MCF7, BT-549, and MDA-MB-453 revealed higher *Jab1 *promoter activity compared to normal mammary epithelial cells MCF-10A, HMEC and 184A (Figure 2c). Jab1 protein levels were also found to be higher in the panel of breast cancer cells compared with the normal mammary epithelial cells HMEC and MCF-10A (Figure 2d). Therefore, the transcription factors responsible for promoting *Jab1 *transcription are either not present in normal cells or not as active as in tumor cells. Another possibility is that normal cells may express transcription suppressors acting on this region. In Figure 2b, the activity of the deletion constructs demonstrated differing ratios of luciferase activity between MCF7 and MCF-10A suggesting that different dominant regulatory factors may exist in each of these cell lines. However, interestingly, the same differential in promoter activity was seen between the -472 and -344 constructs in both cell lines.

### Identification of C/EBP and GATA transcription elements located in the -472/-345 region of the Jab1 promoter

Our data suggest that the -472 to -345 (-472/-345) region is key to driving the transcription of *Jab1*. We therefore sought to identify the transcription factors that regulated *Jab1 *through this region. Using the TRANSFAC database, we identified a number of putative transcription factor binding sites in the -472/-345 region, including sites for C/EBP (-444/CAAC/-441) and GATA-1 (-435/TATCT/-431) (Figure 1b). Because these transcription factors are activated in some cases of tumorigenesis [24,25], we speculated that they contributed to the increased transcription of *Jab1*.

C/EBPs are a family of basic leucine-zipper transcription factors, including C/EBP-α, C/EBP-β, -∂, -ε, -γ, and -ζ [26]. Of these, C/EBP-α, -β, and -∂ are expressed in the mammary gland, with C/EBP-β proposed to play a role in breast cancer [27,28]. The GATA family of transcription factors is required for erythroid and megakaryocytic differentiation [29]. Unlike other GATA members, GATA-1 has not been associated with any solid tumors, but mutations in *GATA-1 *are associated with essentially all cases of acute megakaryoblastic leukemia in children with Down syndrome [30].

**Figure 3:**
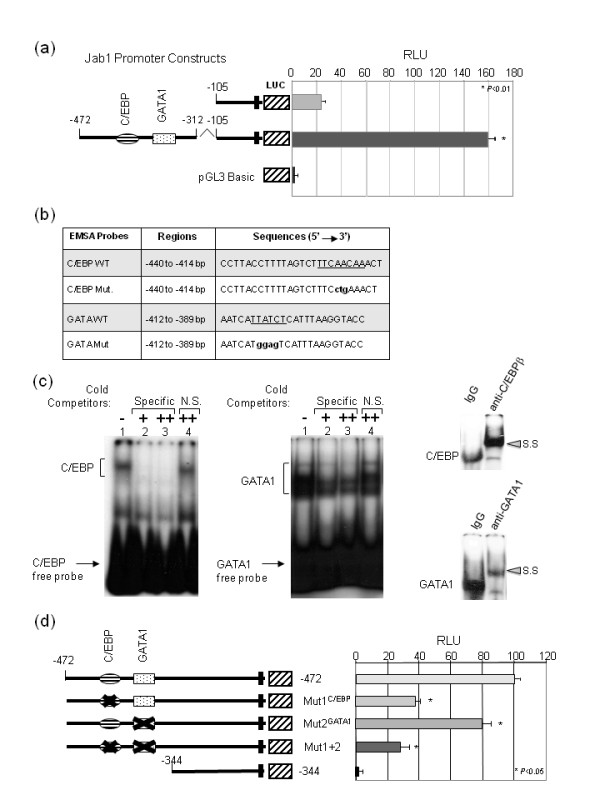
**Functional analysis of the *Jab1 *promoter**. (**a**) The -472/-312 region contains a regulatory sequence that is sufficient to activate transcription of the *Jab1 *promoter when cloned in front of the TATA and CAAT boxes. MCF7 cells were transfected with either the -105/+83-Luc or -472/-312, and -105/+83 Jab1-Luc constructs and subjected to luciferase reporter assays. Values were compared with the -105 Jab1-Luc reporter by Student's t test, * *P *< 0.01. (**b**) Sequence of the probes used in EMSA assays. The probes span the sequence from -440 to -414 or from -412 to -389 of the *Jab1 *promoter and encompass the C/EBP or GATA sites. (**c**) Binding of C/EBP and GATA-1 to the Jab1 promoter. EMSAs showing binding of nuclear proteins C/EBP and GATA-1 to the C/EBP and GATA-1 binding sites of the *Jab1 *promoter. End-labeled oligonucleotide probes corresponding to the -440/-414 (C/EBP) or -413/-389 (GATA-1) regions of the WT *Jab1 *promoter sequence were incubated with nuclear extract proteins from MCF7 cells and separated on a 4.5% polyacrylamide gel. Lane 1 shows binding between probe and nuclear extract. Specificity was determined by addition of 50 and 100 molar excess of unlabeled specific cold probe, and mutant probe as specific and nonspecific (NS) competitors as indicated. Lane 4 shows complexes observed in the presence of probes containing mutations in C/EBP or GATA-1. Supershift of the complex was observed following incubation with C/EBPβ or GATA-1 antibodies as shown in the right panels. (**d**) MCF7 cells were transfected with the -472 Jab1-Luc, -472 C/EBP mutant (Mut1 ^C/EBP^), -472 GATA-1 mutant (Mut 2 ^GATA1^), Mut1+2, or -344 Jab1-Luc plasmids. Mutation of the C/EBP(Mut1^C/EBP^) and GATA-1 (Mut2^GATA1^) binding sites reduced *Jab1 *promoter activity by about 60% and 20%, respectively, and by 75% when mutated together. The firefly luciferase activity of each sample was normalized to that of *Renilla *luciferase (pRL). Data present the mean ± standard deviation of three independent experiments. All values were compared with the -472 Jab1-Luc reporter by Student's t test. C/EBP, CCAAT/enhancing binding protein; EMSA, electrophoretic mobility shift assay; Jab1, c-Jun activation domain-binding protein-1; NS, nonspecific binding; SS, supershift.

To pinpoint the functional significance of the C/EBP and GATA-1 binding sites detailed within the *Jab1 *promoter we performed promoter and EMSA analysis of these regions. Cloning of this region in front of the -105/+83 sequence containing the TATA and CAAT boxes was sufficient to drive *Jab1 *promoter activity (Figure 3a). To determine whether these putative elements are capable of binding transcription factors, we performed a series of EMSA experiments with nuclear extracts from MCF7 breast cancer cells. The oligonucleotides and mutants for the C/EBP and GATA-1 binding sites are shown in Figure 3b. The -462/436 and -435/-417 probes showed transcription factor binding activity to the DNA containing the C/EBP and GATA-1 binding sites, respectively (Figure 3c). The cold specific oligonucleotides competed efficiently for binding, whereas a cold mutant competitor containing a mutation in the C/EBP or GATA-1 binding site did not. Further, a supershift was observed when the oligonucleotides were incubated with antibodies to C/EBP-β and GATA-1 (Figure 3c).

To assess whether any of these sites is important for expression of Jab1, each was mutated individually in luciferase reporter plasmids. We introduced mutations in the -472/-345 region of the *Jab1 *promoter to disrupt C/EBP and GATA-1 binding and compared their activity with the -472 Jab1-Luc promoter construct in transient transfection assays. Mutation of either C/EBP or GATA-1 binding sequence reduced *Jab1 *promoter activity by approximately 40% and 20%, respectively, and mutation of both sites resulted in a reduction of approximately 75% (Figure 3d). Interestingly, C/EBP and GATA binding sites homology from the human and mice promoter regions were found very well conserved [see Figure S1 in Additional file 1].

### C/EBP-α, C/EBP-β, and GATA-1 transactivate the Jab1 promoter

**Figure 4:**
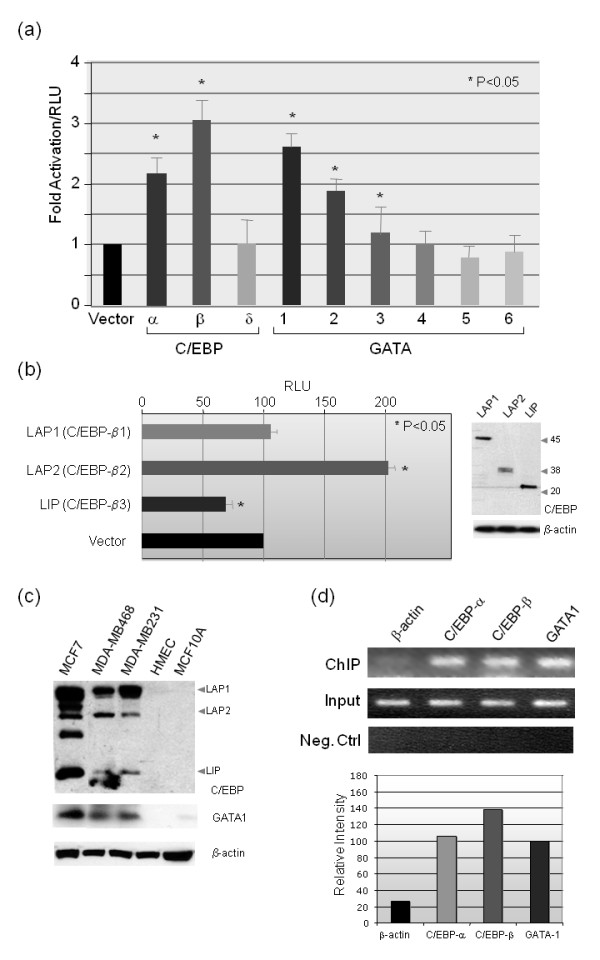
**C/EBP-α, C/EBP-β and GATA-1 transactivate the *Jab1 *promoter**. (**a**) -472/+83 Jab1-Luc reporter plasmid was cotransfected with expression plasmids for C/EBP-α, C/EBP-β, C/EBP-δ, and GATA-1-6. The results are presented as fold activation compared with cells transfected with pcDNA3.1 vector control. All values were compared with the vector control by Student's t test. (**b**) Left, LAP1 (C/EBP-β1), LAP2 (C/EBP-β2), or LIP (C/EBP-β3) plasmids, along with the -474 Jab1-Luc promoter construct, were cotransfected with the *Renilla *luciferase plasmid pRL-null (transfection control) into MCF7 cells, and luciferase activity was assayed. Right, western blot analyses to demonstrate the protein expression of LAP1, LAP2 and LIP. (**c**) Endogenous expression of the C/EBPβ isoforms (LAP1, LAP2, and LIP) and GATA-1 were detected at higher levels in breast cancer cell lines compared with normal mammary epithelial cells by western blot analysis. **(d) **Identification of proteins in complexes by ChIP assays. Antibodies to β-actin, C/EBP-α, C/EBP-β, or GATA-1 were used to immunoprecipitate protein/DNA complexes from the sonicated lysates of MCF7 cells. After reversing the cross-linking, DNA was precipitated and PCR was performed using primers to amplify the -472/-344 promoter DNA. As a negative control, primers for the upstream promoter region -642/-475 were used. Bottom panel represents quantification of the data with ImageJ software. C/EBP, CCAAT/enhancing binding protein; ChIP, chromatin immunoprecipitation; Jab1, c-Jun activation domain-binding protein-1; LAP, liver-enriched activating protein; LIP, liver-enriched inhibitory protein; PCR, polymerase chain reaction; pRL, *Renilla *luciferase reporter vector; RT-PCR, reverse transcription polymerase chain reaction.

Next, we examined which of the C/EBP and GATA family members are important for *Jab1 *promoter activity. We cotransfected different members of the C/EBP and GATA family, C/EBP-α, C/EBP-β, or C/EBP-δ, or GATA1-6, into MCF7 cells, along with the -472 Jab1-Luc plasmid (Figure 4a). Compared with control cells transfected with vector alone, cells transfected with C/EBP-α, C/EBP-β, or GATA-1 showed the greatest increase in -472 Jab1-Luc reporter activity (2.2-, 3.0-, and 2.6-fold, respectively). GATA-2 and GATA-3 also showed a significant increase in activity but for the purpose of this study were not studied in detail.

Interestingly, the transcription factor C/EBP-β has been associated with breast cancer [27,31]. It is translated into three different isoforms: C/EBP-β1, a 55-kDa liver-enriched activating protein (LAP1); C/EBP-β2, a 42- to 46-kDa protein also called LAP2 and; C/EBP-β3 a 20-kDa liver-enriched inhibitory protein (LIP) [25,32]. Of the three C/EBP-β isoforms, LAP2 has been specifically observed in breast cancer, and overexpression of this isoform can induce epithelial-mesenchymal transition [28]. LIP, however, is unable to activate gene transcription, but is still able to bind to DNA and dimerize, and therefore acts as a dominant negative [27]. It has been suggested that the LAP/LIP ratio may be an important indicator of C/EBP-β transcription [25]. We found that LAP2 activates the *Jab1 *promoter (over two-fold), whereas LAP1 had little effect and LIP decreased activity by about 16% (Figure 4b). Further, analysis of the C/EBPβ isoforms in breast cancer cells compared with normal, revealed higher levels of the LAP-1 and -2 isoforms in MCF7, MDA-MB-468 and MDA-MB-231 breast cancer cells compared with normal mammary epithelial cells HMEC and MCF-10A (Figure 4c). Interestingly, the LIP expression was also expressed at higher levels in the breast cancer cells compared with normal (Figure 4c). This in line with the increased endogenous Jab1 expression detected in these breast cancer cells compared with the normal mammary epithelial cells as shown in Figure 2d. We also detected higher GATA-1 expression in the breast cancer cells compared with normal mammary epithelial cells and taken together could be a driving force in leading Jab1 expression in breast cancer.

### C/EBP-α, C/EBP-β, and GATA-1 bind to the Jab1 promoter

To further determine whether C/EBP isoforms and GATA-1 bind to the *Jab1 *promoter, we performed ChIP assays on chromatin obtained from MCF7 cells (Figure 4d). When chromatin was incubated with β-actin, no product was observed. When chromatin was incubated with antibodies to C/EBP-α, C/EBP-β, and GATA-1 and the -472/-344 region was amplified by PCR, a product was observed. These data suggest that C/EBP-α, C/EBP-β, and GATA-1 bind specifically to the -472/-344 region of the *Jab1 *promoter.

### Stat3 enhances the activity of C/EBP-α and C/EBP-β on the Jab1 promoter

**Figure 5:**
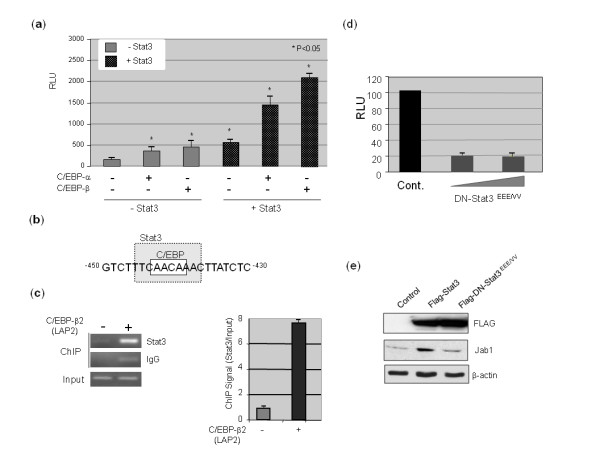
**Stat3 can synergize with C/EBP-α and C/EBP-β to transactivate the *Jab1 *promoter**. (**a**) The -472/+83 Jab1-Luc reporter plasmid was cotransfected with expression plasmids for C/EBP-α, or C/EBP-β. Plasmid amounts were normalized by the addition of empty expression plasmid. Following transfection, MCF7 cells were incubated for another 36 hours. The results are presented as fold activation compared with cells transfected with pcDNA vector control. (**b**) A potential STAT-binding element (TTN5AA consensus sequence) overlaps the C/EBP binding site (CAAC). (**c**) Binding of Stat3 to the Jab1 promoter is enhanced by the addition of C/EBP. MDA-MB-231 cells were transfected with either control of C/EBP-β2 and collected for ChIP assays using an antibodies against Stat3 and primers for the -472/-344 region of the Jab1 promoter. Right panel represents quantification of the band intensities and normalized to the input with ImageJ software. (**d**) MDA-MB-468 cells were cotransfected with either pcDNA control, a Flag-tagged Stat3 (Flag-Stat3), or Flag-tagged dominant negative (DN)-Stat3^EEE/VV^, along with the -472 Jab1-Luc construct, and subjected to luciferase reporter assays. (**e**) Overexpression of Flag-tagged Stat3 increased Jab1 protein levels while a reduction was seen with Flag-DN- Stat3^EEE/VV^. C/EBP, CCAAT/enhancing binding protein; ChIP, chromatin immunoprecipitation; Jab1, c-Jun activation domain-binding protein-1.

The C/EBP transcription factors form heterodimers with other C/EBP family members and other transcription factors. We observed that *Jab1*-driven luciferase reporter activity increased upon addition of conditioned medium to MCF7 cells (data not shown). One of the pathways activated by conditioned medium is the Stat3 pathway, which has also been linked with breast tumorigenesis [33]. Moreover, Stat3 has been shown to interact with the C/EBP transcription factors and increase their activity [24]. We therefore investigated whether Stat3 can transactivate the *Jab1 *promoter, alone or in combination with C/EBP-α or C/EBP-β. Stat3 alone increased *Jab1 *promoter activity, but Stat3 along with C/EBP-α and C/EBP-β synergistically increased *Jab1 *promoter activity (Figure 5a), suggesting that Stat3 interacts with C/EBP on the *Jab1 *promoter. To that end, we observed a STAT general consensus site (TTN5AA) [34] that overlaps the C/EBP site within the *Jab1 *promoter sequence (-472 to -345) and was not identified by transcription factor database searches (Figure 5b). Stat1, Stat3, Stat4, and Stat92E all bind to this generalized consensus site [34,35].

We next performed a ChIP assay to determine whether Stat3 could bind with C/EBP-β2 co-operatively to the *Jab1 *promoter using MDA-MB-231 cells, which have constitutively activated Stat3. Since C/EBP-β2 (or LAP2) seems to be a major activator of the *Jab1 *promoter (Figure 4), we next focused only on C/EBP-β2. Binding of Stat3 to the *Jab1 *promoter was increased greater than seven-fold when C/EBP-β2 was transfected into the cells (Figure 5c). Taken together, these data suggest that C/EBP-β2 and Stat3 bind to the *Jab1 *promoter to increase Jab1 promoter activities.

We further investigated whether inhibition of Stat3 affects *Jab1 *promoter activity in MDA-MB-468 cells, which have constitutively activated Stat3. Expression of a dominant-negative mutant of Stat3 reduced over 80% *Jab1 *promoter activity (Figure 5d). The EEE/VV mutation renders the protein incapable of DNA binding [36]. Expression of exogenous wild type Stat3 increased Jab1 expression, whereas the dominant-negative EEE/VV mutation reduced Jab1 protein levels (Figure 5e**)**.

### Inhibition of Stat3 and Src decrease Jab1 promoter activity and protein expression

**Figure 6:**
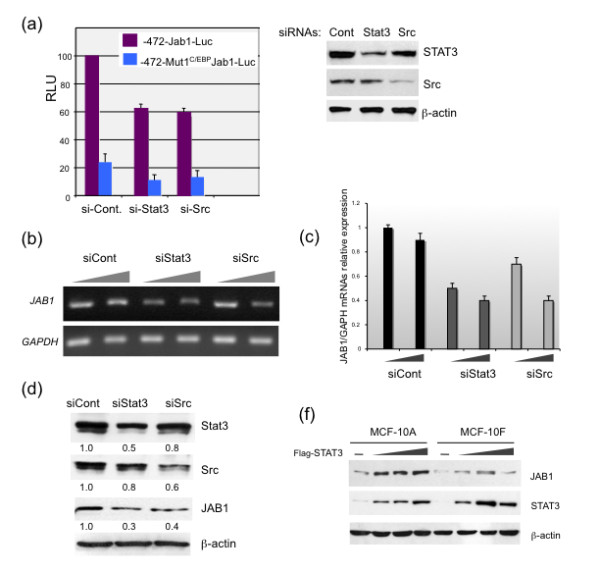
**Inhibition of Stat3 and Src by siRNA reduces *Jab1 *promoter activity in breast carcinoma cells**. (**a**) MDA-MB-468 cells were cotransfected with siRNA targeting Stat3 (siStat3) or Src (siSrc), along with either the -472 Jab1-Luc construct or -472-Mut1^C/EBP ^Jab1-Luc (as a control) and subjected to luciferase reporter assays, western blot analyses to demonstrate the efficient knockdown of Stat3 and Src protein levels are shown in the right panel. (**b**) MDA-MD-468 cells were transfected with increasing doses of siStat3 and siSrc and Jab1 was detected by RT-PCR, quantification of Jab1 RNA levels is shown in (**c**). (**d**) Western blot analysis demonstrates that Jab1 protein levels decreased following inhibition of Stat3 and Src by siRNA. Jab1, c-Jun activation domain-binding protein-1; Mut, mutation; siRNA, small interfering RNA; RT-PCR, reverse transcription polymerase chain reaction; STAT, signal transducer and activator of transcription.

To further demonstrate the regulation of Jab1 by Stat3, we examined the effect of inhibition of Stat3 and also its upstream activator Src using siRNA [37,38]. Inhibition of Stat3 and Src by siRNA resulted in a dramatic reduction of *Jab1 *promoter activity (Figure 6a), mRNA levels (Figures 6b and 6c), and Jab1 protein expression (Figure 6d). Similar results were obtained with MDA-MB-231 cells (data not shown). Collectively, these results demonstrate that the Src/Stat3 signaling pathway plays an important role in the regulation of *Jab1 *transcription.

### Stat3-induced Jab1 transcriptional activation and protein expression

**Figure 7:**
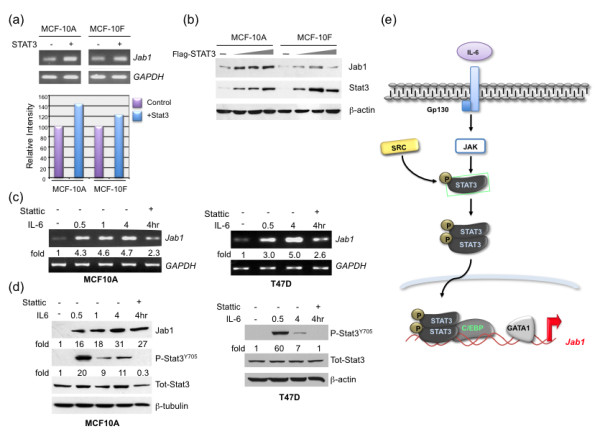
**Stat3 pathway contributes to Jab1 transcriptional activation and increased protein levels**. (**a**) Increased expression of Stat3 in the normal mammary epithelial MCF-10A cells increased Jab1 RNA levels as detected by RTPCR, quantification of Jab1 RNA levels is shown in the bottom panel. (**b**) Ectopic expression of Flag-Stat3 (0, 1, 2, and 3 μg) in normal mammary epithelial cells (MCF-10A and MCF-10F) resulted in increased Jab1 protein levels. (**c**) Treatment with the cytokine IL-6 increased Jab1 mRNA levels - seen by RT-PCR - in both normal MCF-10A and breast cancer T47D cells that were blocked by the Stat3 inhibitor Stattic. (**d**) Treatment with IL-6 increased Jab1 protein levels following activation of Stat3 (P-Stat3 Y705) that was inhibited by the addition of Stattic. (**e**) Schematic representation of the major molecular mechanism of Stat3-induced transcriptional activation of Jab1 through two signaling axes - Src/Stat3 and IL-6/Stat3. Jab1, c-Jun activation domain-binding protein-1; RT-PCR, reverse transcription polymerase chain reaction; STAT, signal transducer and activator of transcription.

To determine the biological significance of Stat3-mediated Jab1 expression, the role of Stat3 in normal human breast epithelial cells was investigated. As Jab1 expression in normal mammary epithelial cells is low, we asked whether overexpression of Stat3 could enhance *Jab1 *transcription in these cells. Ectopic expression of Stat3 in normal mammary epithelial cells (MCF-10A and MCF-10F) resulted in increased Jab1 mRNA and protein levels (Figures 7a and 7b). Therefore, the Stat3 transcription factor is in part responsible for promoting *Jab1 *transcription in breast cancer cells and is either not present or not as active in the MCF-10A and MCF-10F cells.

We further investigated whether an upstream activator of Stat3, the cytokine IL-6, could be driving increased Jab1 expression. Treatment with IL-6 for 30 minutes increased phosphorylation of Stat3 on tyrosine 705 and resulted in increased Jab1 mRNA (Figure 7c) and protein levels (Figure 7d) within the short time of 30 minutes, 1 hour, and 4 hours that was partially blocked by the addition of the Stat3 inhibitor Stattic. The same trends were observed in the breast cancer cell line T47D and the mammary epithelial cells, MCF-10A. IL-6 also resulted in increased *Jab1 *promoter activity in MCF7 and T47D cells [See Figure S2 in Additional file 1].

Taken together, it is evident that both IL-6 and Src signaling through Stat3 is contributing to *Jab1 *transcription and increased expression in breast cancer. Further, it is possible that Stat3 and C/EBPβ could be binding to the Jab1 promoter either separately or together to mediate increased *Jab1 *transcriptional activity. A proposed model for the activation of *Jab1 *transcription is shown in Figure 7e.

## Discussion

Jab1 is commonly overexpressed in patients with breast cancer as well as other tumor types. The mechanism by which Jab1 is regulated is currently not known and our data suggest that this may occur at the transcriptional level. In this study, the *Jab1 *promoter was analyzed to identify the molecular basis of *Jab1 *gene expression and to give insight into the mechanisms by which Jab1 is overexpressed in cancer. *Jab1 *promoter analysis led to the identification of C/EBP-β, GATA-1, and Stat3 as positive regulators of *Jab1 *transcription in breast cancer cells. Promoter deletion studies identified a region between -472 and -345 that has significant transcriptional activity, as evidenced by the dramatic reduction in luciferase reporter activity when this region has been deleted (Figure 2a). Mutation of both the C/EBP and GATA-1 sites together resulted in decreased luciferase reporter activity of approximately 75% when combined. We identified C/EBP, GATA-1, and Stat3 consensus sequences located in this region and their binding was confirmed by EMSA and ChIP assays. We established that C/EBP, GATA-1, and Stat3 are positive regulators of *Jab1 *promoter activity. As these transcription factors are activated during tumorigenesis, and because Jab1 is overexpressed in a number of tumors, we demonstrate that these transcription factors indeed increase transcription of *Jab1*.

In our study, we identified C/EBP as a potential transcriptional activator for *Jab1*, specifically C/EBPα and C/EBPβ-2. C/EBPβ-1 is expressed in normal breast cells while expression of C/EBPβ-2 is known to be expressed specifically in invasive primary breast tumor samples or cells lines [39]. Of the three isoforms of C/EBP-β, the transactivating form of LAP2 resulted in a two-fold increase in *Jab1 *transcriptional activity while the inhibitor isoform LIP decreased activity. C/EBP-β appears to play a critical role in the development of both the mammary gland and cancers therein through its involvement in development, differentiation, and proliferation of mammary epithelial cells [25,27,31]. As Jab1 is frequently upregulated in breast cancer, it is possible that LAP2 is a major factor in driving *Jab1 *transcription during the tumorigenic process. Of note, our study detected higher levels of all three C/EBP-β isoforms in a panel of breast cancer cell lines compared with normal mammary epithelial cells (Figure 4d), which is contrary to previous studies that identified mainly higher expression of LAP2 in breast cancer. Yet, LAP2 was the isoform that resulted in the greatest increase in transcriptional activity of Jab1. This increased expression could certainly be driving increased Jab1 activity in breast cancer cells.

Further, we identified a co-existing Stat3 binding site within the C/EBP binding site on the *Jab1 *promoter. Ectopic overexpression of Stat3 increased transcriptional activity as well as mRNA and protein levels of Jab1. This was further increased with overexpression of the activated form of Stat3. Constitutive activation of Stat3 occurs commonly in cancer, including breast cancer and has been demonstrated to contribute to tumorigenic processes [40]. Stat3 can mediate signaling through upstream receptor tyrosine kinases such as epidermal growth factor receptor (EGFR) and platelet-derived growth factor receptor (PDGR) as well as upstream non-receptor tyrosine kinases such as Abl and Src-related kinases. These receptors are constitutively activated in cancer, typically through genetic alterations [40]. The oncogenic Src protein kinase itself is overexpressed in a large number of tumor types and interacts with multiple tyrosine kinase receptors, including EGFR and HER2 [41] to mediate its oncogenic effects of promoting growth and metastasis. We found that Src, an activator of Stat3, is involved in *Jab1 *transcription. Overexpression of both Stat3 and Src in normal mammary epithelial cells resulted in increased Jab1 mRNA and protein levels. These data provide the first evidence that Jab1 is a direct downstream target of Stat3 and Src. Additionally, inhibition of Src by siRNA reduced *Jab1 *promoter activity in a manner similar to inhibition of Stat3. We further identified one upstream activator of Stat3, IL-6, that mediated activation of Jab1 expression.

Since the present study began, Jab1 expression has been linked to the HER2 signaling pathway. HER2 has been found to stimulate *Jab1 *transcriptional activity in NIH3T3 cells stably expressing the HER2 receptor [21]. This stimulation took place through the AKT/β-catenin/TCF-4 signaling pathway in breast cancer cells overexpressing the HER2 receptor. The TCF binding site is in the same area as our region of interest, between -472 and -344. In our laboratory, we also found overexpression of Jab1 in NIH3T3 and MCF7 cells that stably express the HER2 receptor (data not shown). However, inhibition of this pathway by the anti-HER2 antibody trastuzumab (Herceptin) or AKT inhibitors in MCF7 and SKBR3 cells did not reduce *Jab1 *promoter activity. However, trastuzumab did inhibit Jab1 protein levels in BT-474 breast cancer cells as well as phosphorylation of AKT and Stat3 (data not shown). The regulation of Jab1 expression by HER2 through the AKT pathway is of great interest, and further studies could strengthen our understanding on the role of Jab1 in the tumorigenic process.

As overexpression of Jab1 is frequently observed in breast cancer, further investigation of the pathways that modulate *Jab1 *transcription would provide insight into the role Jab1 plays in the tumorigenic process therein. Activation of the Stat3 pathway in breast cancer can occur through many pathways, including those of EGFR, HER2, IL-6 receptors, IL-11 receptors, and progesterone receptors [42]. Experimental activation of these pathways, followed by evaluation of *Jab1 *promoter activity and mRNA levels, could provide insight into the mechanisms by which *Jab1 *transcription is activated. Our data provide evidence of activation of *Jab1 *transcription through IL-6 and Src mediated activation of Stat3 as shown in Figure 7e. It is possible that other activators upstream of Stat3 could be mediating this downstream effect as well and warrants further investigation.

## Conclusions

In summary, the present study demonstrates that the Src/Stat3 and C/EBP signaling pathways positively regulate the expression of the Jab1 oncogene. Our results show that Stat3 and LAP2 (C/EBP-β2) are the two major transcription factors that contribute to Jab1 overexpression that leads to increased proliferation of breast cancer cells. Our findings reveal a novel mechanism of Jab1 regulation and provide functional and mechanistic links between two major signaling axes - Src/Stat3 and IL-6/Stat3 - that are involved in controlling Jab1 oncogenic protein activation. Understanding the mechanisms by which Jab1 expression is deregulated may help in the development of drugs that target additional key elements responsible for this important deregulation.

## Abbreviations

C/EBP: CCAAT/enhancing binding protein; ChIP: chromatin immunoprecipitation; CRL: cullin-RING-ubiquitin ligase; CSN: COP9 signalosome; CUL-1: cullin 1; DMEM: Dulbecco's modified Eagle's medium; EGFR: epidermal growth factor receptor; EMSA: electrophoretic mobility shift assay; FBS: fetal bovine serum; Jab1: c-Jun activation domain-binding protein-1; JAMM: Jab1 MPN domain metalloenzyme; HER: human epidermal growth factor receptor; HIF-1α: hypoxia inducible factor-1 alpha; LAP: liver-enriched activating protein; LIP: liver-enriched inhibitory protein; MIF: macrophage migration inhibitory factor; PCR: polymerase chain reaction; PDGR: platelet-derived growth factor receptor; RT-PCR: reverse-transcription polymerase chain reaction; siRNA: small interfering RNA; STAT: signal transducer and activator of transcription; TCF: T-cell transcription factor.

## Competing interests

The authors declare that they have no competing interests.

## Authors' contributions

TJS carried out all of the studies and drafted the manuscript. QZ assisted in many aspects of the study including the execution of deletion analysis and siRNA analysis. LT contributed to the cloning of Jab1 promoter and its analysis. VTT and AMB participated in cell culture, siRNA, and western blotting experiments. ALK provided plasmid constructs, assisted in the primer extension analysis and participated in study design. XFL and WSL, participated in the study design, and provided important intellectual support. FXC conceived the study, participated in its design, coordination and interpretation of the results and finalized the manuscript. All authors read and approved the final manuscript.

## Supplementary Material

Additional file 1 **Figure S1**. C/EBP and GATA binding sites homology from the human and mice promoter regions. **Figure S2**. IL-6 mediated activation of Jab1 promoter activity. Click here for file

## References

[B1] Claret FX Hibi M Dhut S Toda T Karin M A new group of conserved coactivators that increase the specificity of AP-1 transcription factors Nature 1996 383 453 457 10.1038/383453a0 8837781

[B2] Schwechheimer C Deng XW COP9 signalosome revisited: a novel mediator of protein degradation Trends Cell Biol 2001 11 420 426 10.1016/S0962-8924(01)02091-8 11567875

[B3] Chamovitz DA Segal D JAB1/CSN5 and the COP9 signalosome. A complex situation EMBO Rep 2001 2 96 101 10.1093/embo-reports/kve028 11258719 PMC1083824

[B4] Shackleford TJ Claret FX JAB1/CSN5: a new player in cell cycle control and cancer Cell Div 2010 5 26 10.1186/1747-1028-5-26 20955608 PMC2976740

[B5] Cope G Suh GS Aravind L Schwarz SE Zipursky SL Koonin EV Deshaies RJ Role of predicted metalloprotease motif of Jab1/Csn5 in cleavage of Nedd8 from Cul1 Science 2002 298 608 611 10.1126/science.1075901 12183637

[B6] Wei N Serino G Deng XW The COP9 signalosome: more than a protease Trends Biochem Sci 2008 33 592 600 10.1016/j.tibs.2008.09.004 18926707

[B7] Tomoda K Kubota Y Kato J Degradation of the cyclin-dependent-kinase inhibitor p27Kip1 is instigated by Jab1 Nature 1999 398 160 165 10.1038/18230 10086358

[B8] Bech-Otschir D Kraft R Huang X Henklein P Kapelari B Pollmann C Dubiel W COP9 signalosome-specific phosphorylation targets p53 to degradation by the ubiquitin system Embo J 2001 20 1630 1639 10.1093/emboj/20.7.1630 11285227 PMC145508

[B9] Oh W Lee EW Sung YH Yang MR Ghim J Lee HW Song J Jab1 induces the cytoplasmic localization and degradation of p53 in coordination with Hdm2 J Biol Chem 2006 281 17457 17465 10.1074/jbc.M601857200 16624822

[B10] Wan M Cao X Wu Y Bai S Wu L Shi X Wang N Jab1 antagonizes TGF-beta signaling by inducing Smad4 degradation EMBO Rep 2002 3 171 176 10.1093/embo-reports/kvf024 11818334 PMC1083965

[B11] Bae MK Ahn MY Jeong JW Bae MH Lee YM Bae SK Park JW Kim KR Kim KW Jab1 interacts directly with HIF-1alpha and regulates its stability J Biol Chem 2002 277 9 12 11707426 10.1074/jbc.C100442200

[B12] Wei N Deng XW The COP9 signalosome Annu Rev Cell Dev Biol 2003 19 261 286 10.1146/annurev.cellbio.19.111301.112449 14570571

[B13] Sui L Dong Y Ohno M Watanabe Y Sugimoto K Tai Y Tokuda M Jab1 expression is associated with inverse expression of p27(kip1) and poor prognosis in epithelial overian tumors Clin Cancer Res 2001 7 4130 4135 11751512

[B14] Kouvaraki MA Tian L Mansouri A Zhang Q Kumar R Kittas C Claret FX Jun activation domain-binding protein 1 expression in breast cancer inversely correlates with the cell cycle inhibitor p27(Kip1) Cancer Res 2003 63 2977 2981 12782606

[B15] Rassidakis GZ Claret FX Lai R Zhang Q Sarris AH McDonnell TJ Medeiros LJ Expression of p27(Kip1) and c-Jun activation binding protein 1 are inversely correlated in systemic anaplastic large cell lymphoma Clin Cancer Res 2003 9 1121 1128 12631617

[B16] Shintani SLC Mihara M Hino S Nakashiro K Hamakawa H Skp2 and Jab1 expression are associated with inverse expression of p27(KIP1) and poor prognosis in oral squamous cell carcinomas Oncology 2003 65 355 362 10.1159/000074649 14707456

[B17] Dong Y Sui L Watanabe Y Yamaguchi F Hatano N Tokuda M Prognostic significance of Jab1 expression in laryngeal squamous cell carcinomas Clin Cancer Res 2005 11 259 266 15671554

[B18] Richardson KS Zundel W The emerging role of the COP9 signalosome in cancer Mol Cancer Res 2005 3 645 653 10.1158/1541-7786.MCR-05-0233 16380502 PMC2943958

[B19] Adler AS Lin M Horlings H Nuyten DS van de Vijver MJ Chang HY Genetic regulators of large-scale transcriptional signatures in cancer Nat Genet 2006 38 421 430 10.1038/ng1752 16518402 PMC1435790

[B20] Adler AS Littlepage LE Lin M Kawahara TL Wong DJ Werb Z Chang HY CSN5 isopeptidase activity links COP9 signalosome activation to breast cancer progression Cancer Res 2008 68 506 515 10.1158/0008-5472.CAN-07-3060 18199546 PMC2646416

[B21] Hsu MC Chang HC Hung WC HER-2/neu transcriptionally activates Jab1 expression via the AKT/beta-catenin pathway in breast cancer cells Endocr Relat Cancer 2007 14 655 667 10.1677/ERC-07-0077 17914096

[B22] Mansouri A Ridgway LD Korapati AL Zhang Q Tian L Wang Y Siddik ZH Mills GB Claret FX Sustained activation of JNK/p38 MAPK pathways in response to cisplatin leads to Fas ligand induction and cell death in ovarian carcinoma cells J Biol Chem 2003 278 19245 19256 10.1074/jbc.M208134200 12637505

[B23] Nikolov DB Burley SK RNA polymerase II transcription initiation: a structural view Proc Natl Acad Sci USA 1997 94 15 22 10.1073/pnas.94.1.15 8990153 PMC33652

[B24] Numata A Shimoda K Kamezaki K Haro T Kakumitsu H Shide K Kato K Miyamoto T Yamashita Y Oshima Y Nakajima H Iwama A Aoki K Takase K Gondo H Mano H Harada M Signal transducers and activators of transcription 3 augments the transcriptional activity of CCAAT/enhancer-binding protein alpha in granulocyte colony-stimulating factor signaling pathway J Biol Chem 2005 280 12621 12629 15664994 10.1074/jbc.M408442200

[B25] Zahnow CA CCAAT/enhancer binding proteins in normal mammary development and breast cancer Breast Cancer Res 2002 4 113 121 10.1186/bcr428 12052253 PMC138725

[B26] Ramji DP Foka P CCAAT/enhancer-binding proteins: structure, function and regulation Biochem J 2002 365 561 575 12006103 10.1042/BJ20020508 PMC1222736

[B27] Grimm SL Rosen JM The role of C/EBPbeta in mammary gland development and breast cancer J Mammary Gland Biol Neoplasia 2003 8 191 204 10.1023/A:1025900908026 14635794

[B28] Bundy LM Sealy L CCAAT/enhancer binding protein beta (C/EBPbeta)-2 transforms normal mammary epithelial cells and induces epithelial to mesenchymal transition in culture Oncogene 2003 22 869 883 10.1038/sj.onc.1206216 12584567

[B29] Lowry JA Mackay JP GATA-1: one protein, many partners Int J Biochem Cell Biol 2006 38 6 11 10.1016/j.biocel.2005.06.017 16095949

[B30] Crispino JD GATA1 in normal and malignant hematopoiesis Semin Cell Dev Biol 2005 16 137 147 10.1016/j.semcdb.2004.11.002 15659348

[B31] Zahnow CA CCAAT/enhancer-binding protein beta: its role in breast cancer and associations with receptor tyrosine kinases Expert Rev Mol Med 2009 11 e12 19351437 10.1017/S1462399409001033 PMC3095491

[B32] Singh SP Lipman J Goldman H Ellis FH Aizenman L Cangi MG Signoretti S Chiaur DS Pagano M Loda M Loss or altered subcellular localization of p27 in Barrett's associated adenocarcinoma Cancer Res 1998 58 1730 1735 9563491

[B33] Clevenger CV Roles and regulation of stat family transcription factors in human breast cancer Am J Pathol 2004 165 1449 1460 10.1016/S0002-9440(10)63403-7 15509516 PMC1618660

[B34] Horvath CM Wen Z Darnell JE Jr A STAT protein domain that determines DNA sequence recognition suggests a novel DNA-binding domain Genes Dev 1995 9 984 994 10.1101/gad.9.8.984 7774815

[B35] Xu X Sun YL Hoey T Cooperative DNA binding and sequence-selective recognition conferred by the STAT amino-terminal domain Science 1996 273 794 797 10.1126/science.273.5276.794 8670419

[B36] Kaptein A Paillard V Saunders M Dominant negative stat3 mutant inhibits interleukin-6-induced Jak-STAT signal transduction J Biol Chem 1996 271 5961 5964 10.1074/jbc.271.11.5961 8626374

[B37] Yu CL Meyer DJ Campbell GS Larner AC Carter-Su C Schwartz J Jove R Enhanced DNA-binding activity of a Stat3-related protein in cells transformed by the Src oncoprotein Science 1995 269 81 83 10.1126/science.7541555 7541555

[B38] Reddy EP Korapati A Chaturvedi P Rane S IL-3 signaling and the role of Src kinases, JAKs and STATs: a covert liaison unveiled Oncogene 2000 19 2532 2547 10.1038/sj.onc.1203594 10851052

[B39] Eaton EM Hanlon M Bundy L Sealy L Characterization of C/EBPbeta isoforms in normal versus neoplastic mammary epithelial cells J Cell Physiol 2001 189 91 105 10.1002/jcp.1139 11573208

[B40] Yu H Jove R The STATs of cancer--new molecular targets come of age Nat Rev Cancer 2004 4 97 105 10.1038/nrc1275 14964307

[B41] Irby RB Yeatman TJ Role of Src expression and activation in human cancer Oncogene 2000 19 5636 5642 10.1038/sj.onc.1203912 11114744

[B42] Aggarwal BB Sethi G Ahn KS Sandur SK Pandey MK Kunnumakkara AB Sung B Ichikawa H Targeting signal-transducer-and-activator-of-transcription-3 for prevention and therapy of cancer: modern target but ancient solution Ann N Y Acad Sci 2006 1091 151 169 10.1196/annals.1378.063 17341611

