# Supplementary material for: Stat3 and CCAAT/enhancer binding protein beta (C/EBP-beta) regulate Jab1/CSN5 expression in mammary carcinoma cells
Source: Breast Cancer Res. 2011 Jun 20;13(3):R65. doi: 10.1186/bcr2902 (PMC3218954; doi:10.1186/bcr2902)

Supplemental Figure Legends

Figure S1. C/EBP and GATA binding sites homology from the human and mice promoter regions. The sequences of the 5'-flanking regions of the human and mouse Jab1 promoter were aligned within the C/EBP and GATA regions using the ClustalW sequence alignment program. Homology regions within C/EBP and GATA binding sites are shaded. Sequence identities are indicated with a (*). For the mouse Jab1 sequence location, the translational start site was set at +1.

Figure S2. IL-6 mediated activation of Jab1 promoter activity. Briefly, MCF-7 or T47D were co-transfected with -472-Jab1-Luc reporter construct and pRL (Renilla luciferase) for 24 hours. The following day, the cells were treated with or without IL-6 (Invitrogen) for indicated time points before harvesting for read-out using luciferase assay (Promega) as being described in Materials and Methods.

Figure S1.

```
human Jab1-C/EBP(-428/-413)    AGTCTTTCAACAAAC
mouse Jab1-C/EBP(-508/-493)    AGTATTCCAAACAAT
                               *** ** ***  **

human Jab1-GATA1(-414/-398)    TTATCTCATTTAAGGTA
mouse Jab1-GATA1(-493/-477)    TTATTTCGTTTGAAGCA
                               **** ** *** * * *
```

Figure S2.

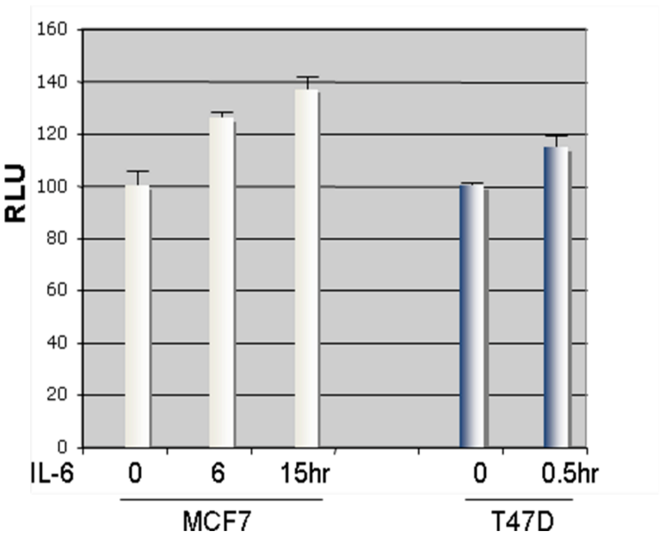

Supplement: Additional file 1 — Figure S1. C/EBP and GATA binding sites homology from the human and mice promoter regions. Figure S2. IL-6 mediated activation of Jab1 promoter activity. [file bcr2902-S1.PDF]
